# Supplementary material for: Inflammatory factors and risk of lung adenocarcinoma: a Mendelian randomization study mediated by blood metabolites
Source: Front Endocrinol (Lausanne). 2024 Aug 27;15:1446863. doi: 10.3389/fendo.2024.1446863 (PMC11384989; doi:10.3389/fendo.2024.1446863)
Supplement: Supplementary file 1 [file DataSheet1.zip › supplementary files/S2/GCST90200389/forest.pdf]

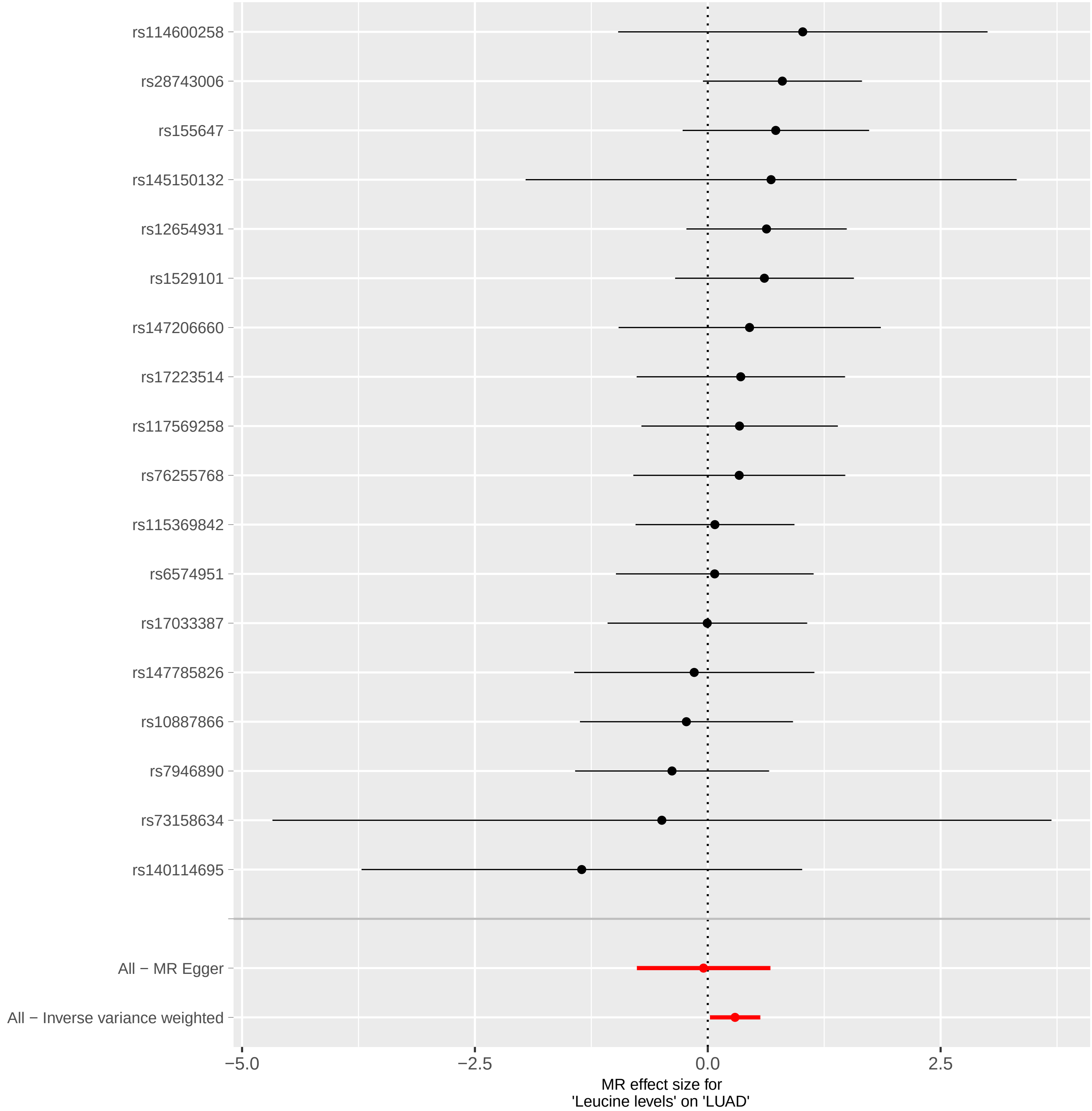

rs114600258
rs28743006
rs155647
rs145150132
rs12654931
rs1529101
rs147206660
rs17223514
rs117569258
rs76255768
rs115369842
rs6574951
rs17033387
rs147785826
rs10887866
rs7946890
rs73158634
rs140114695
All − MR Egger
All − Inverse variance weighted
−5.0
−2.5
0.0
2.5
MR effect size for
'Leucine levels' on 'LUAD'
